# eHealth literacy was associated with anxiety and depression during the COVID-19 pandemic in Nigeria: a cross-sectional study

**DOI:** 10.3389/fpubh.2023.1194908

**Published:** 2023-06-22

**Authors:** Oluwadamilare Akingbade, Khadijat Adeleye, Oluwadamilola Agnes Fadodun, Israel Opeyemi Fawole, Jiaying Li, Edmond Pui Hang Choi, Mandy Ho, Kris Yuet Wan Lok, Janet Yuen Ha Wong, Daniel Yee Tak Fong, Oluwabunmi Ogungbe

**Affiliations:** ^1^The Nethersole School of Nursing, The Chinese University of Hong Kong, Shatin, Hong Kong SAR, China; ^2^Institute of Nursing Research, Osogbo, Osun, Nigeria; ^3^University of Massachusetts, Amherst, MA, United States; ^4^Faculty of Health Sciences, University of Lethbridge, Lethbridge, AB, Canada; ^5^Ladoke Akintola University of Technology, Ogbomoso, Nigeria; ^6^School of Nursing, The University of Hong Kong, Hong Kong, Hong Kong SAR, China; ^7^School of Nursing and Health Studies, Hong Kong Metropolitan University, Hong Kong, Hong Kong SAR, China; ^8^Johns Hopkins University School of Nursing, Baltimore, MD, United States

**Keywords:** eHealth literacy, COVID-19, pandemic preparedness, depression, anxiety, psychological outcomes, Nigeria, mental health—state of emotional and social well-being

## Abstract

**Background:**

Electronic health (eHealth) literacy may play an important role in individuals’ engagement with online mental health-related information.

**Aim:**

To examine associations between eHealth literacy and psychological outcomes among Nigerians during the Coronavirus disease-2019 (COVID-19) pandemic.

**Methods:**

This was a cross-sectional study among Nigerians conducted using the ‘COVID-19’s impAct on feaR and hEalth (CARE) questionnaire. The exposure: eHealth literacy, was assessed using the eHealth literacy scale, and psychological outcomes were assessed using the PHQ-4 scale, which measured anxiety and depression; and the fear scale to measure fear of COVID-19. We fitted logistic regression models to assess the association of eHealth literacy with anxiety, depression, and fear, adjusting for covariates. We included interaction terms to assess for age, gender, and regional differences. We also assessed participants’ endorsement of strategies for future pandemic preparedness.

**Results:**

This study involved 590 participants, of which 56% were female, and 38% were 30 years or older. About 83% reported high eHealth literacy, and 55% reported anxiety or depression. High eHealth literacy was associated with a 66% lower likelihood of anxiety (adjusted odds ratio aOR, 0·34; 95% confidence interval, 0·20–0·54) and depression (aOR: 0·34; 95% CI, 0·21–0·56). There were age, gender, and regional differences in the associations between eHealth literacy and psychological outcomes. eHealth-related strategies such as medicine delivery, receiving health information through text messaging, and online courses were highlighted as important for future pandemic preparedness.

**Conclusion:**

Considering that mental health and psychological care services are severely lacking in Nigeria, digital health information sources present an opportunity to improve access and delivery of mental health services. The different associations of e-health literacy with psychological well-being between age, gender, and geographic region highlight the urgent need for targeted interventions for vulnerable populations. Policymakers must prioritize digitally backed interventions, such as medicine delivery and health information dissemination through text messaging, to address these disparities and promote equitable mental well-being.

## 1. Introduction

Coronavirus Disease 2019 (COVID-19) pandemic has had a considerable impact on physical and psychosocial health (1). and is an emerging risk factor for chronic diseases such as hypertension, diabetes mellitus, cardiovascular disease, and chronic kidney disease (2), including debilitating post-acute sequelae of COVID-19 (3, 4). While COVID-19 adversely impacts physical health, a range of psychological issues has been linked to the pandemic as both direct and indirect impacts. The prevalence of psychological stress, insomnia, and psychological distress following COVID-19 has been reported to be between 20% and 30% globally (5). Similarly, in Nigeria, various studies have reported a range of psychological issues among Nigerians during the pandemic, including anxiety, depression, insomnia and inadequate social support (6–9). Risk factors associated with increased psychological distress during the pandemic include younger age group (≤40 years), female gender, previous mental health problems, unemployment, student status, and frequent exposure to social media or news related to COVID-19 (10).

Electronic health (eHealth) includes health-related services and information delivered or enhanced through the internet or related health technologies (11), including the capacity to evaluate health information obtained from electronic sources and use what is learned to address or resolve a health issue (12). The COVID-19 pandemic has increased global eHealth and mobile health (mHealth) usage alongside substantial increases in screen time (13). In Nigeria, the use of digital devices significantly increased during the COVID-19 pandemic (14). Nigeria has the largest mobile market in Africa, with 199.6 million mobile connections as of March 2022 (15); hence, mobile phones have been a resource for seeking health information in Nigeria as well as in many low and middle-income countries (16).

COVID-19 poses critical challenges to the utility of eHealth literacy, for which the World Health Organization and other agencies warned strongly against infodemics; “an overabundance of information and rapid spread of misleading and fabricated news, images, and videos, which, like the virus, is highly contagious, grows exponentially, and undermines public health measures and leads to unnecessary loss of life” (17). The exceptionally high volume and rapid evolution of COVID-19 pandemic-related information, with a proliferation of misinformation and disinformation, contributed to widespread public confusion and can have severe and lethal health and social consequences, further eroding trust in science (18). There have been speculations that these could contribute to increased anxiety, psychological stress, suicidal ideation, and worsened mental health (19). However, high eHealth literacy also offers opportunities for rapid dissemination of information and may contribute to assured safety and help people make better health-related decisions (20).

Healthy eHealth literacy and internet use may be linked to better psychological wellbeing. Previous reports have shown reports of a negative correlation between eHealth literacy and depression, insomnia, and post-traumatic disorder (21). Improving eHealth literacy has been recommended to address psychological distress (22). The mental health of Nigerians was adversely affected during the pandemic (9, 23). With the high internet and social media use in Nigeria, examining eHealth literacy during the COVID-19 pandemic and its contribution to psychological outcomes is important. This is vital to planning and preparedness for communication and mitigation strategies in future pandemics or crises. Hence, this study aimed to: assess the associations between eHealth literacy and psychological outcomes among Nigerians during the COVID-19 pandemic; examine the effect modification of age, gender, and geographic differences on these associations; and investigate residents’ preferences for future preparations.

We hypothesized that there would be no significant association between eHealth literacy and anxiety, depression, and fear of COVID-19, after adjusting for age, gender, education and employment.

## 2. Methods

### 2.1. Study design and population

This cross-sectional study was conducted in Nigeria as part of a larger international cross-sectional study on societal perceptions of COVID-19’s impact and preferences for future preparations. As cross-sectional designs help gain insight into population characteristics and behaviors at a given time (24), it was deemed fit for this study as the researchers were interested in investigating eHealth usage among Nigerians during the pandemic. The STROBE cross-sectional reporting guidelines were followed in reporting this study. The study was approved by the Institutional Review Board of the University of Hong Kong/ Hospital Authority Hong Kong West Cluster (UW 20-272). Additional details of the study have been reported in the published protocol (25). Participants were recruited from the six geopolitical zones in Nigeria: North Central, North East, North West, South East, South South, and South West through an online survey using both convenience and snowball sampling methods.

### 2.2. Sampling, recruitment and data collection

The inclusion criteria for the study include Nigerians who could understand English. Although Nigeria has over 250 ethnic groups, with each ethnic group having indigenous languages, English is the national language of communication (26). Similarly, participants with access to the internet and who use social media platforms were included. This is because evidence suggests increased internet and social media usage during the COVID-19 pandemic (14). Persons less than 18 years, non-Nigerians, Nigerians who were not residing in the country at the time of data collection, and those who were cognitively impaired were excluded from the study. The sample size was calculated based on the estimation of the prevalence of a health-related issue. A conservative scenario of 50%, with a 5% margin of error in a 95% CI, required 385 subjects (25). However, data collection continued until 590 respondents were recruited to provide a broader representation.

Social media platforms were the preferred recruitment methods to reach participants across the six geopolitical zones, specifically WhatsApp, Facebook, Twitter, LinkedIn, and Instagram. Participants were recruited from various tertiary institutions and National Youth Service Corp (NYSC) camps across various regions in Nigeria to facilitate representation (27). Initially, we conveniently sampled participants and then snowballed by encouraging participants to share the survey with their friends and family. This sampling and recruitment strategy was adopted due to the COVID-19 pandemic that restricted access to participants in person. This strategy also improved access to a large population of participants as people could participate in the survey within the comfort of their geographical location. The tertiary institutions and National Youth Service Corp (NYSC) camps comprise Nigerians from different geographical regions, age groups, gender, ethnic and cultural groups. Participants were encouraged to share the survey with their friends and family. Participants who agreed to participate were required to consent before they were given access to complete the survey. For every survey participant, HK$1 (about US$0.13) was donated to the Red Cross for each completed questionnaire in the respondent’s area. The data collection period spanned 3 months.

Data collection was conducted using the ‘COVID-19’s impAct on feaR and hEalth (CARE) questionnaire (28), launched on the Qualtrics platform. The instrument has been validated, and the psychometric properties have been presented in the study protocol (25). For the Nigerian survey, a contextually relevant validation question was added: “What is the capital of Lagos?” to enhance internal validity; the survey was also pilot tested to ensure consistency and understanding of survey items. The online survey included a captcha to ensure that the respondents were actual participants, not automated users or bots. Data collection was conducted between January and March 2021. The questionnaire sections included sociodemographic characteristics with eight items age, gender, marital status, occupational status, perceived social status, pregnancy status (if applicable), and household size. The fear scale had eight items on a 5-point Likert scale; higher total scores indicate a higher fear level. This scale was adapted from a previous study conducted in Nigeria with a Cronbach’s alpha of 0.90 (29). The e-health literacy scale (eHEALS) had eight items that assessed electronic sources and channels of information-seeking behavior concerning the COVID-19 outbreak on a 5-point Likert scale, exposure to and pursuit of various types of health-related information, perception of the credibility, accuracy, and usefulness of the information, and confidence in locating the accurate information. The reliability of the eHEALS has been confirmed in a previous study conducted in Nigeria, with a Cronbach’s alpha of 0.92 (30). The PHQ-4 scale had two items that measured anxiety, and the other two measured depression on a 4-point Likert scale. Higher scores indicate a higher level of anxiety and depression. The reliability of the PHQ-4 scale has been confirmed in a previous study among Nigerian University students with a good test–retest reliability score (r = 0.894, *p* < 0.001) (31). Participants were also asked to rank the most important preparation for future pandemics; these were; online consultation with doctors (e.g., Zoom, Skype), instant personalized health advice by online chatbot, telephone health advice, online courses, instant streaming courses (e.g., Zoom, Skype), receiving health information through email, receiving health information through text messaging (e.g., SMS, WhatsApp), receiving health information from social media (e.g., Facebook, Instagram, Twitter), receiving health information from mobile app, get medicine prescribed in a hospital visit/follow-up in a community pharmacy, medicine delivery, online shopping, food delivery. Other sections of the questionnaire included lifestyle and health-related impact of COVID-19. The development and validation of the instrument have been published (25).

### 2.3. Statistical analysis

Data were analyzed using Stata Statistical package and R Programming. Data were meticulously organized and underwent thorough quality control procedures to ensure its accuracy and integrity, including checks for missing responses, duplicates, and inconsistencies. Missing data was determined to be missing at random (MAR) and were excluded from the final analyses. A sum score of the eHEALS scale was obtained and dichotomized into “low” and “high” using a ≥ 26 cut point (32). Psychological outcomes, anxiety, and depression were derived from the PHQ-4 scale; for anxiety—a sum score of the first two items of the PHQ-4 scale was obtained and dichotomized into “no anxiety” and “anxiety” using a cut point of ≥3. Similarly, for depression, a sum score of the last two items on the PHQ-4 scale was obtained and dichotomized into “no depression” and “depression” using a cut point of ≥3 (33). A sum score of the fear of COVID scale was also derived and dichotomized using a ≥ 16.5 cut point (34). Descriptive statistics were summarized using frequencies and percentages or means and standard deviations as applicable. We described participants’ characteristics stratifying by eHealth Literacy levels, gender, age, and region. We fitted logistic regression analyses to assess the association between eHealth literacy (predictor) and psychological outcomes, specifically depression, anxiety, and fear of COVID-19, adjusting for age, gender, education, and employment as covariates. We conducted subgroup analyses using logistic regression with interaction terms to assess the differences in the association between eHealth literacy and psychological outcomes by age, gender, geographical region, and healthcare worker status. A *post hoc* Bonferroni correction was used to adjust for multiple comparisons; Bonferroni thresholds for each subgroup analysis was set by dividing the alpha level (0.05) by the number of pairwise tests. Descriptive statistics were also used for participants’ responses to the most important preparations for future pandemics, displaying this in a Likert chart.

### 2.4. Ethical considerations

Ethical approval was sought and obtained for the study, which has been reported in the study protocol (20). The authors respected all ethical obligations by providing online information about the research as well as consent forms. Participants were asked to ascertain if they understood the content of the informed consent by indicating the same online. Prior to accessing the online questionnaire, participants were asked to indicate whether they were willing to proceed or not proceed with the survey. Participants were clearly reminded of their rights to voluntary participation. On no account was a participant forced to participate in the study. Also, data privacy and confidentiality were ensured per IRB regulations and national ethical guidelines. All data were stored on password-protected servers compliant with national privacy laws.

## 3. Results

### 3.1. Sample characteristics

Participant characteristics stratified by eHealth literacy level are shown in Table 1. This study involved 590 participants, of which 56% were female, 38% were 30 years or older, 63% had at least a bachelor’s degree, 53% were employed, and 54% lived in the Southwest region. For outcomes of depression and anxiety, 55% reported depression, and 55% reported anxiety. Participants with high health literacy were more likely to be female, have at least a bachelor’s degree, be employed, and live in the country’s Southwestern region. There were gender, age, and regional differences in the sample (Supplementary Tables S1, S2).

**Table 1:** Participant characteristics, stratified by eHealth literacy level.

Characteristics	Total	eHealth literacy		*p*-value
		Low	High	
	*N* = 590	*N* = 103	*N* = 487	
BMI, M(±SD)	19·0 (3·4)	18·9 (2·5)	19·1 (3·6)	0.710
Gender, *n* (%)				**0.014**
Male	255 (43·2)	56 (54·4)	199 (40·9)	
Female	331 (56·1)	47 (45·6)	284 (58·3)	
Missing	4 (0·7)	0 (0·0)	4 (0·8)	
Age category, *n* (%)				0.470
18–24 years	171 (29·0)	25 (24·3)	146 (30·0)	
25–29 years	255 (43·2)	49 (47·6)	206 (42·3)	
≥30 years	164 (27·8)	29 (28·2)	135 (27·7)	
Education category, *n* (%)				**0.002**
<Bachelors	219 (37·1)	52 (50·5)	167 (34·3)	
≥Bachelors	371 (62·9)	51 (49·5)	320 (65·7)	
Employment category, *n* (%)				**<0.001**
Not employed	277 (46·9)	64 (62·1)	213 (43·7)	
Employed	313 (53·1)	39 (37·9)	274 (56·3)	
Marital, *n* (%)				0.200
Married/cohabitation/common-law	156 (26·4)	27 (26·2)	129 (26·5)	
Separated/divorced/widowed	7 (1·2)	3 (2·9)	4 (0·8)	
Single	427 (72·4)	73 (70·9)	354 (72·7)	
Region, *n* (%)				**0.004**
North Central	78 (13·2)	19 (18·4)	59 (12·1)	
North East	20 (3·4)	6 (5·8)	14 (2·9)	
North West	37 (6·3)	12 (11·7)	25 (5·1)	
South East	22 (3·7)	6 (5·8)	16 (3·3)	
South South	45 (7·6)	11 (10·7)	34 (7·0)	
South West	317 (53·7)	41 (39·8)	276 (56·7)	
Missing	71 (12·0)	8 (7·8)	63 (12·9)	
Depression, *n* (%)				**<0.001**
No	266 (45·1)	24 (23·3)	242 (49·7)	
Yes	266 (45·1)	79 (76·7)	245 (50·3)	
Anxiety, *n* (%)				**<0.001**
No	265 (44·9)	24 (23·3)	241 (49·5)	
Yes	325 (55·1)	79 (76·7)	246 (50·5)	
Health care professional, *n* (%)				**<0.001**
No	276 (46·8)	80 (77·7)	196 (40·2)	
Yes	314 (53·2)	23 (22·3)	291 (59·8)	

Bold *p* < 0.05.

### 3.2. eHealth literacy and psychological outcomes

About 83% of the sample had high eHealth literacy. Higher eHealth literacy was associated with 66% lower odds of both depression (adjusted odds ratio aOR: 0·34; 95% confidence interval, 0.21–0.56) and anxiety (aOR, 0.34; 95%CI, 0.20–0.54), after accounting for age, gender, education, and employment. There were no observed statistical associations between eHealth literacy and fear of COVID-19 (Table 2).

**Table 2:** Associations between eHealth literacy and psychological outcomes.

Psychological outcomes	Prevalence, *n* (%)	Odds ratio (95% confidence interval)	
		Unadjusted	Adjusted^†^
Depression	266 (45·1)	**0·31 (0·19–0·50)**	**0·34 (0·21–0·56)**
Anxiety	325 (55·1)	**0·31 (0·19–0·51)**	**0·34 (0·20–0·54)**
Fear of COVID-19	499 (84·58)	0·68 (0·36–1·30)	0·68 (0·35–1·33)

Bold: *P* < 0·05.

† Adjusted for age, gender, education, employment.

**Figure 1:**
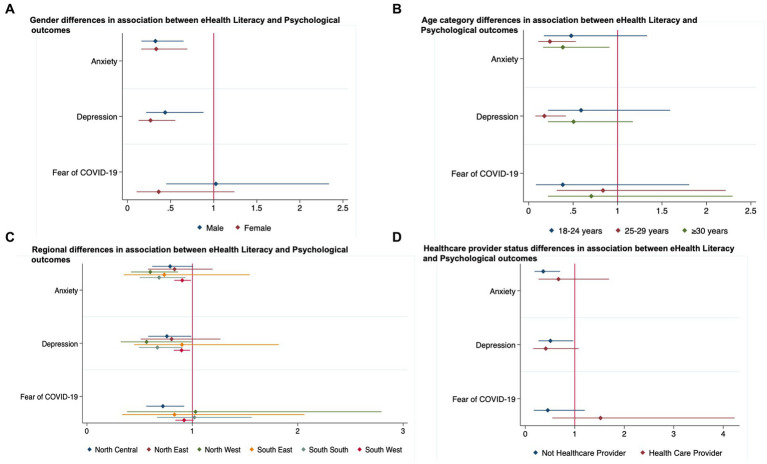
**(A–D)** Age, gender and geographical region and healthcare worker status differences in the association between eHealth Literacy and Psychological outcomes.

**Figure 2:**
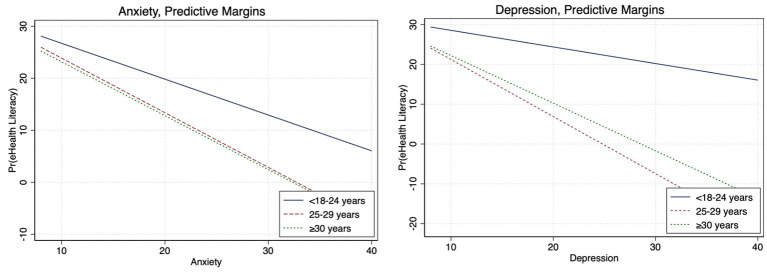
Adjusted gender differences between eHealth literacy and anxiety and depression (Bonferroni adjusted).

We observed differences in the associations between eHealth literacy and psychological outcomes by age, gender, and geographical region (Supplementary Tables S4–S6). After accounting for covariates, among men, high eHealth literacy was associated with 56% lower odds of depression (aOR, 0.44; 95%CI, 0.22–0.88) and 68% lower odds of anxiety (aOR, 0.32; 95%CI, 0.16–0.65), while for women, high eHealth literacy was linked to 87% lower odds of depression (aOR, 0.27; 95%CI, 0.13–0.55) and 67% lower odds of anxiety (aOR, 0.33; 95%CI, 0.11–0.24; Figure 1A; Supplementary Table S4). The Bonferroni corrected margins plot demonstrates the probability of anxiety and depression by eHealth literacy by gender (Figure 2).

**Figure 3:**
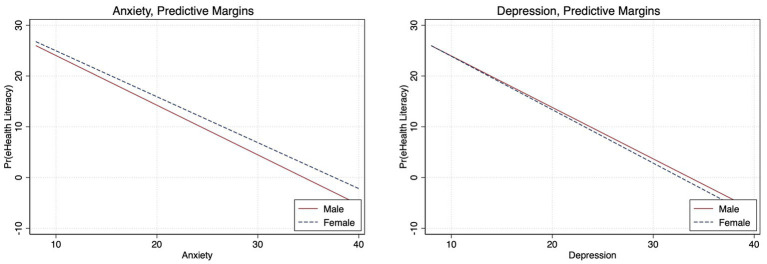
Adjusted age differences between eHealth literacy and anxiety and depression (Bonferroni adjusted).

High eHealth literacy was not associated with depression, anxiety, or fear of COVID-19 among persons aged 18–24 years; however, among those aged 25–29 years, there was 82% (aOR, 0.18; 95%CI, 0.07–0.42) and 76% (aOR, 0.24; 95%CI, 0.11–0.53) lower likelihood of depression and anxiety, respectively. Among persons older than 30 years, the odds of anxiety were 62% (aOR, 0.38; 95%CI, 0.16–0.91) lower for those with high eHealth literacy compared to those with low eHealth literacy (Figures 1B; Supplementary Table S5). The Bonferroni corrected margins plot demonstrates the probability of anxiety and depression by eHealth literacy by age category (Figure 3).

**Figure 4:**
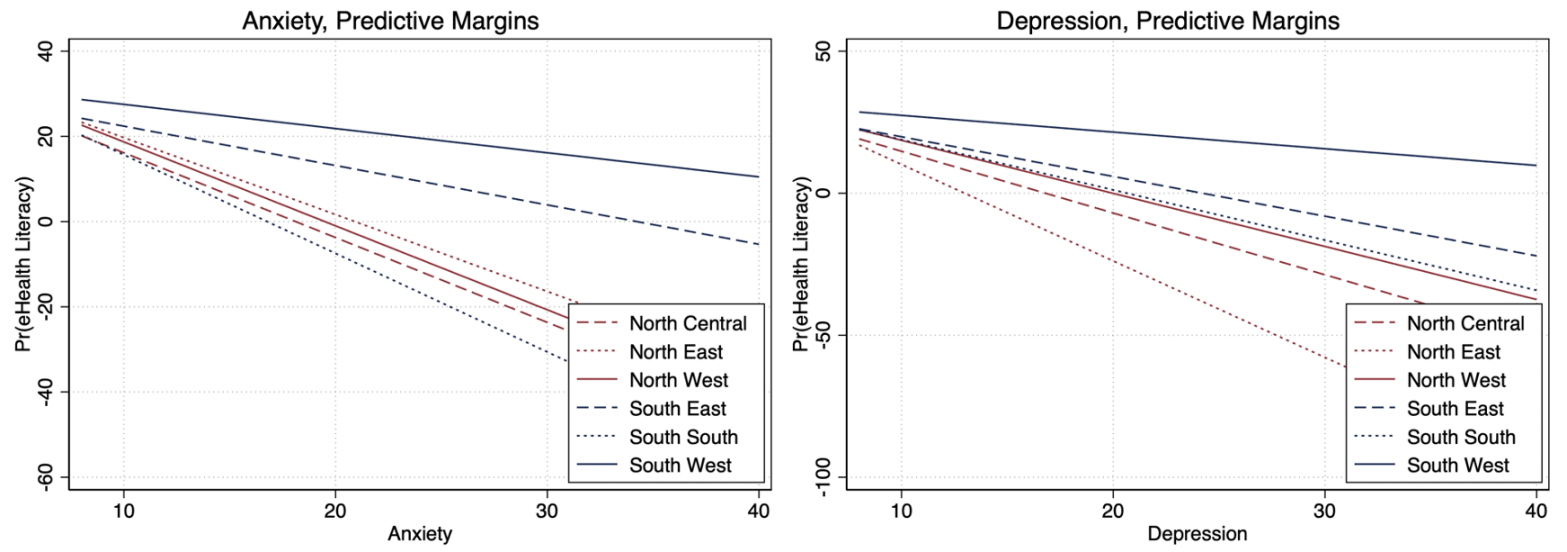
Adjusted regional differences between eHealth literacy and anxiety and depression (Bonferroni adjusted).

Among participants living in the Northcentral region of the country, high eHealth literacy was associated with 22% (aOR, 0.78; 95%CI, 0.58–0.99) lower odds of depression and 28% (aOR, 0.72; 95%CI, 0.56–0.92) lower odds of fear of COVID-19. For those living in the Northwest, high eHealth literacy was linked to 43% (aOR, 0.57; 95%CI, 0.32–0.99) and 40% (aOR, 0.60; 95%CI, 0.42–0.86) lower odds of depression and anxiety, respectively. Persons living in the South region with high eHealth literacy were 33% (aOR, 0.67; 95%CI, 0.49–0.91) and 32% (aOR, 0.68; 95%CI, 0.51–0.93) less likely to report depression and anxiety respectively; while the odds of depression (aOR, 0.90; 95%CI, 0.82–0.99) and anxiety (aOR, 0.90; 95%CI, 0.83–0.99) was 10% lower for persons living in the Southwest with high eHealth literacy (Figure 1C; Supplementary Table S6). There were no differences in the associations between eHealth literacy and psychological outcomes by healthcare worker status (Figure 1D). The Bonferroni corrected margins plot demonstrates the probability of anxiety and depression by eHealth literacy by region (Figure 4).

### 3.3. eHealth-related strategies for future pandemic preparedness

**Figure 5:**
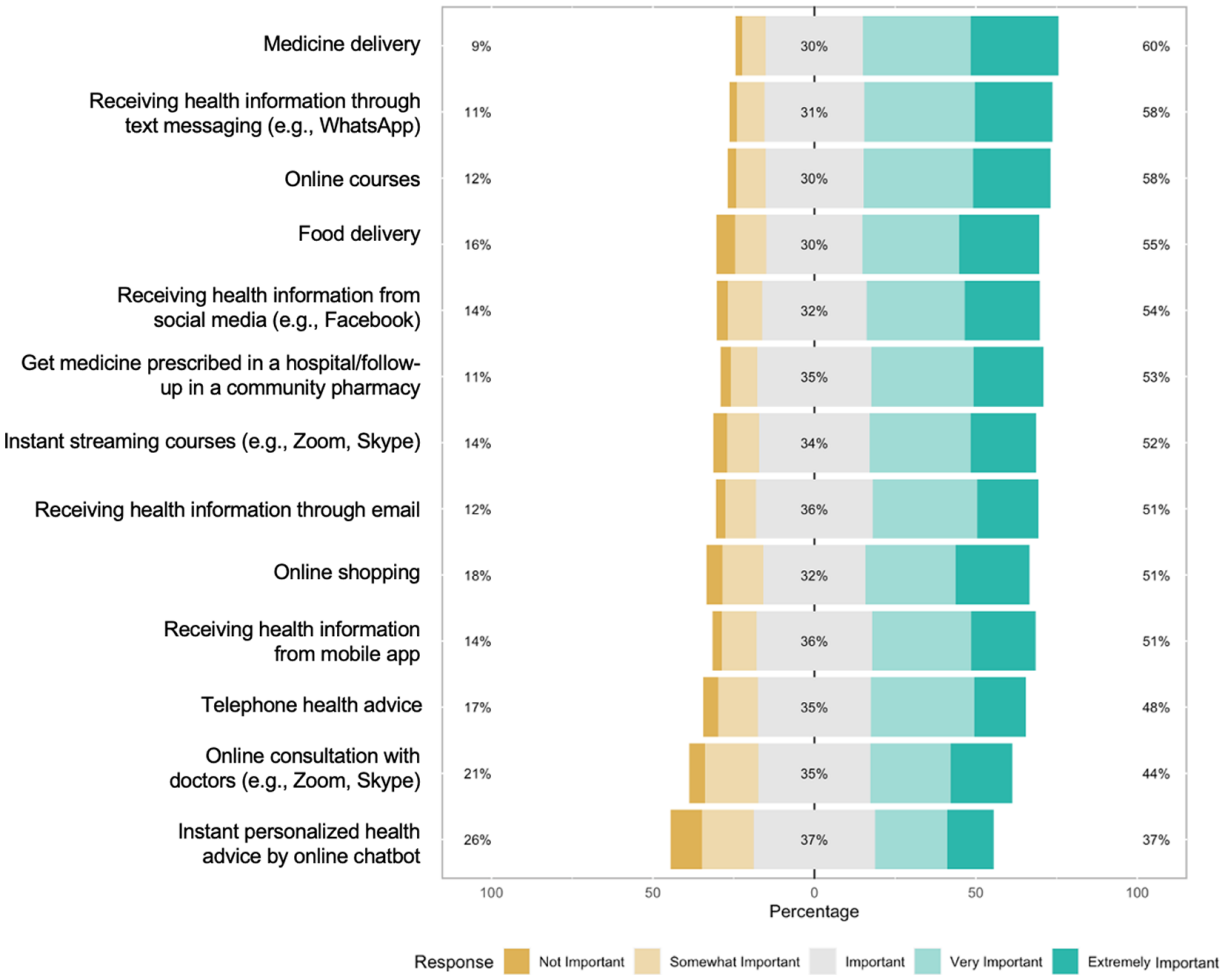
Participants’ endorsement of eHealth-related strategies for future pandemic preparedness.

Many of the strategies were rated important (Figure 5). The pandemic preparedness strategies rated to be most important by the participants were medicine delivery (extremely/very important, 60%; important, 30%), receiving health information through text messaging (extremely/very important, 58%; important, 31%), online courses (extremely/very important,58%; important, 30%), food delivery (extremely/very important, 55%; important, 30%), and receiving health information from social media (extremely/very important, 54%; important, 32%; Figure 2).

## 4. Discussion

We examined the association between eHealth literacy and psychological outcomes during the COVID-19 pandemic in Nigeria. Our study showed five key findings; first, majority of our participants have high eHealth literacy levels. Second, the prevalence of self-reported anxiety and depression is also substantial. Third, high eHealth literacy was associated with a lower likelihood of anxiety and depression, and fourth, there are age, gender, and geographical differences in the association between eHealth literacy and psychological outcomes. Finally, eHealth-related strategies such as medicine delivery, receiving health information through text messaging, and online courses were highlighted as important strategies for future pandemic preparedness (Figure 5). These findings have important implications for improving mental health services through digital technologies in Nigeria.

Our findings on high eHealth literacy can be explained by the increased mobile phone usage in Nigeria reported during the pandemic (14, 35). This is similar to the results from various countries that reported increased usage of mobile phones during the pandemic (36–38). Our results also show that participants with high eHealth literacy were more likely to be female, have at least a bachelor’s degree, be employed and live in the Southwestern region of the country. This is congruent with previous results conducted in Southwest, Nigeria, where women were shown to have a higher literacy level compared to men (39). This is similar to the results of a study conducted in Turkey which found that women had higher levels of eHealth literacy than men because they felt confident and more competent while searching for online information (40). This study found regional differences in eHealth literacy in Nigeria, where Southwest has higher eHealth literacy than other regions (39). This has also been reported in other studies and may reflect English educational attainment, English language proficiency, higher access to the internet and increased exposure to credible medical information that persons in this region have access to Kuyinu et al. (39).

The prevalence of self-reported anxiety and depression in this study aligns with previous studies that reported a range of psychological issues among Nigerians during the pandemic (6–9). This indicate the need for interventions to address psychological issues among this population. Similarly, as a high e-Health literacy was reported among participants in this study, mobile phones can be considered a platform to deliver such interventions, as evidence has revealed that psychological interventions delivered through mobile phones have beneficial psychological effects (41).

We found an association between higher eHealth literacy and lower odds of both depression and anxiety. Previous studies reported an inverse correlation between eHealth literacy and depression and that improving eHealth literacy may contribute to maintaining good psychological well-being (21, 22). Mental health services are severely sparse in Nigeria, and related stigma persists (42). Hence, it is likely that individuals are accessing online sources of mental well-being information, and this could be harnessed to help manage their anxiety and depression. There were no observed statistical associations between eHealth literacy and fear of COVID-19 in our study; this could be attributed to the country’s heightened public awareness of COVID-19 prevention (43). Additionally, persons with high eHealth literacy levels may be better equipped to access credible health information on COVID-19 and less susceptible to misinformation that may fuel fear and anxiety (44). The high mobile usage in Nigeria and eHealth literacy levels present a critical opportunity to advance mental health awareness and encourage mental health services in Nigeria (45). eHealth literacy-informed interventions may also be harnessed to address other health issues in Nigeria; these include verified information about infectious diseases; self-management of chronic disease through digital means (e.g., hypertension diagnosis and management training through an app). Leveraging high eHealth literacy for improving psychological outcomes in Nigeria could prove an important intervention opportunity.

There were age, gender and geographical differences in the association between eHealth literacy. Among women with high eHealth literacy, the likelihood of anxiety and depression was lower compared to men. This corroborated with other studies that have shown that being female, less than 75 years old and having a higher education are associated with eHealth literacy (22). Reasons for this disparity are unclear and could be explored in future studies. The regional differences observed in our results highlight the need for improving internet access for increased educational attainment and eHealth literacy interventions in other regions of the country outside of the Southwestern region. There was a high proportion of healthcare workers in our sample, and the high eHealth literacy in this group may be leveraged for advanced training of health workers, especially during crises and humanitarian situations.

Results from future pandemic strategies endorsed by participants further support the need for health technology-backed interventions in Nigeria. Medicine delivery, receiving health information through text messaging, online courses, food delivery, and receiving health information through social media were endorsed as strategies important to prepare for future pandemics and crises. These are mostly digital interventions that may significantly contribute to improving the health of Nigerians; partnerships between context experts like healthcare workers and digital content experts may further advance such interventions (45). It is important for these interventions to also cater to persons with lower educational attainment and low eHealth literacy. This may include providing health information through platforms like WhatsApp, which is more prevalent among persons with limited digital literacy in Nigeria. Rapid innovations in digital health technologies that improve healthcare access have shown high efficacy in high-income countries. However, access to these health technologies is not equitable globally, with LMICs like Nigeria experiencing global health disparities at a larger scale. Consequently, there is a need for reciprocal innovation, i.e., bidirectional, and iterative exchange of ideas, resources, and innovations to address shared health challenges across diverse global settings (46).

Our study should be interpreted in the context of these limitations. First, this was a cross-sectional design; hence, there was no temporality. Second, the survey was originally designed in Hong Kong and may not have initially included Nigeria in the original context; however, modifications were made to adapt certain survey items to the Nigerian context. Third, our study’s participants were mostly young adults from the Southwestern region, which may have contributed to the high level of eHealth literacy observed; hence, findings from this study might not be generalizable to Nigerians with low literacy, non-social media users and older adults. Fourth, the survey was administered digitally and may have excluded persons with limited digital literacy. Nevertheless, this study has some strengths. First, to our knowledge, this is one of the first studies to examine the associations between eHealth literacy and anxiety and depression in a Nigerian sample. In addition, we employed various recruitment strategies to ensure that participants from different regions of the country were represented in the sample.

Our findings have important implications for the development of interventions to address the scarcity of mental health services in Nigeria. The high eHealth literacy in Nigeria and high use of smartphones and mobile application makes the Nigerian environment suitable for digital health interventions. Participants-endorsed strategies for preparation for future pandemics are critical policy options that may inform healthcare policies. Strategies such as receiving credible health information through social media platforms. Future intervention strategies could leverage digital tools and platforms to provide remote mental health services and incorporate other chronic conditions. Given the critically low performance and ranking of the Nigeria health system (47, 48), these interventions have a high potential to strengthen the primary health care system, and guarantee access to care. An example could include implementing remote counseling and psychiatry services platforms using mobile apps, and telemedicine platforms, to improve access, availability, and utilization of healthcare services. Such strategies could be multi-pronged to address several conditions at once; for instance, such remote platforms could also include remote monitoring of cardiometabolic conditions (e.g., remote blood pressure monitoring), bi-directional messaging between providers and patients, etc. These digital tools should be co-designed and co-developed with patients, health care providers, health system leaders, policymakers and other stakeholders, and should prioritize simplicity in the design with considerations for persons experiencing barriers such as low eHealth literacy, limited broadband access or smartphones, etc. It is critical that digital interventions address health equity and not contribute to widening the digital divide. Importantly, there is a need for health policies that advance the implementation of telemedicine and digital health interventions in Nigeria and ensure equitable funding of health systems in the different regions of the country to improve access to health services.

## 5. Conclusion

In conclusion, our study showed high eHealth literacy among our sample of Nigerian adults. Self-reported prevalence of anxiety and depression is also considerably high in the face of prevalent economic and structural hardship and limited access to mental health services. High eHealth literacy was associated with psychological outcomes of anxiety and depression. eHealth literacy-informed interventions can be invested in to address several pressing health issues in Nigeria and prepare for future pandemics and health-related crises. The age, gender and regional differences observed present important intervention opportunities for interventions. Additionally, digital solutions focused on medicine delivery, receiving health information through text messaging, online courses, etc., are important health technology-backed intervention opportunities in Nigeria. The Nigerian environment may be suitable for digital health interventions to increase access to mental healthcare services due to the country’s high smartphone usage and eHealth literacy, as shown in this study’s result. Importantly, there is a need for health policies that promote the implementation of telemedicine and digital health interventions in Nigeria and guarantee equitable funding of health systems in the various regions of the country in order to enhance access to health services.

## Data availability statement

The raw data supporting the conclusions of this article will be made available by the authors, without undue reservation.

## Ethics statement

The studies involving human participants were reviewed and approved by University of Hong Kong/Hospital Authority Hong Kong West Cluster (UW 20-272). The patients/participants provided their written informed consent to participate in this study.

## Author contributions

EC, MH, KL, JW, and DF conceived the idea for the study and designed the study. OA, OAF, IF, JL, and OO contributed to recruitment and data collection. JL contributed to data collection and management. OO performed data analyses and visualizations for the country-specific data. OA, KA, OAF, and OO wrote the original draft. All authors contributed to the article and approved the submitted version.

## Conflict of interest

The authors declare that the research was conducted in the absence of any commercial or financial relationships that could be construed as a potential conflict of interest.

## Publisher’s note

All claims expressed in this article are solely those of the authors and do not necessarily represent those of their affiliated organizations, or those of the publisher, the editors and the reviewers. Any product that may be evaluated in this article, or claim that may be made by its manufacturer, is not guaranteed or endorsed by the publisher.
