# Supplementary material for: eHealth literacy was associated with anxiety and depression during the COVID-19 pandemic in Nigeria: a cross-sectional study
Source: Front Public Health. 2023 Jun 22;11:1194908. doi: 10.3389/fpubh.2023.1194908 (PMC10323132; doi:10.3389/fpubh.2023.1194908)
Supplement: Supplementary file 1 [file Data_Sheet_1.PDF]

Supplementary Material

eHealth Literacy was Associated with Anxiety and Depression During the COVID-19 Pandemic in Nigeria

*** Correspondence:** Oluwabunmi Ogungbe; oogungbe3@jh.edu

1 Supplementary Figures and Tables

1.1 Supplementary Tables

Supplemental Table 1. Participants' characteristics, stratified by gender

Characteristics	Total	Gender		*p-value*
		Male	Female	
	N=586	N=255	N=331	
BMI, M(±SD)	19·1 (3·4)	19·5 (3·1)	18·7 (3·6)	**0·010**
Age Category, n (%)				**<0·001**
<18-24 years	169 (28·8)	54 (21·2)	115 (34·7)	
25-29 years	253 (43·2)	102 (40·0)	151 (45·6)	
≥30 years	164 (28·0)	99 (38·8)	65 (19·6)	
Education Category, n (%)				0·96
<Bachelors	219 (37·4)	95 (37·3)	124 (37·5)	
≥Bachelors	367 (62·6)	160 (62·7)	207 (62·5)	
Employment Category, n (%)				0·16
Not Employed	275 (46·9)	128 (50·2)	147 (44·4)	
Employed	311 (53·1)	127 (49·8)	184 (55·6)	
Marital, n (%)				**0·019**
Married/Cohabitation/Common-law	156 (26·6)	82 (32·2)	74 (22·4)	
Separated/Divorced/Widowed	7 (1·2)	4 (1·6)	3 (0·9)	
Single	423 (72·2)	169 (66·3)	254 (76·7)	
Region, n (%)				**<0·001**
North Central	76 (13·0)	40 (15·7)	36 (10·9)	
North East	20 (3·4)	12 (4·7)	8 (2·4)	
North West	37 (6·3)	29 (11·4)	8 (2·4)	
South East	21 (3·6)	8 (3·1)	13 (3·9)	
South South	44 (7·5)	23 (9·0)	21 (6·3)	
South West	317 (54·1)	91 (35·7)	226 (68·3)	
Missing	71 (12·1)	52 (20·4)	19 (5·7)	
Depression, n (%)				**0·003**
No	263 (44·9)	97 (38·0)	166 (50·2)	
Yes	323 (55·1)	158 (62·0)	165 (49·8)	
Anxiety, n (%)				0·46
No	263 (44·9)	110 (43·1)	153 (46·2)	
Yes	323 (55·1)	145 (56·9)	178 (53·8)	
Health care professional, n (%)				**<0·001**
No	272 (46·4)	151 (59·2)	121 (36·6)	
Yes	314 (53·6)	104 (40·8)	210 (63·4)	

Supplemental Table 2. Participant characteristics, stratified by age category

Characteristics	Total	Age Category			*p-value*
		18-24 years	25-29 years	≥30 years	
	N=590	N=171	N=255	N=164	
BMI, M(±SD)	19·0 (3·4)	18·1 (3·4)	19·0 (3·1)	20·1 (3·8)	**<0·001**
Gender Category, n (%)					**<0·001**
Male	255 (43·2)	54 (31·6)	102 (40·0)	99 (60·4)	
Female	331 (56·1)	115 (67·3)	151 (59·2)	65 (39·6)	
Missing	4 (0·7)	2 (1·2)	2 (0·8)	0 (0·0)	
Education Category, n (%)					**<0·001**
<Bachelors	219 (37·1)	93 (54·4)	81 (31·8)	45 (27·4)	
≥Bachelors	371 (62·9)	78 (45·6)	174 (68·2)	119 (72·6)	
Employment Category, n (%)					**<0·001**
Not Employed	277 (46·9)	123 (71·9)	121 (47·5)	33 (20·1)	
Employed	313 (53·1)	48 (28·1)	134 (52·5)	131 (79·9)	
Marital Status					**<0·001**
Married/Cohabitation/Common-law, n (%)	156 (26·4)	5 (2·9)	35 (13·7)	116 (70·7)	
Separated/Divorced/Widowed	7 (1·2)	0 (0·0)	0 (0·0)	7 (4·3)	
Single	427 (72·4)	166 (97·1)	220 (86·3)	41 (25·0)	
Region, n (%)					0·16
North Central	78 (13·2)	26 (15·2)	37 (14·5)	15 (9·1)	
North East	20 (3·4)	3 (1·8)	13 (5·1)	4 (2·4)	
North West	37 (6·3)	8 (4·7)	20 (7·8)	9 (5·5)	
South East	22 (3·7)	5 (2·9)	9 (3·5)	8 (4·9)	
South South	45 (7·6)	7 (4·1)	25 (9·8)	13 (7·9)	
South West	317 (53·7)	108 (63·2)	139 (54·5)	70 (42·7)	
Missing	71 (12·0)	14 (8·2)	12 (4·7)	45 (27·4)	
Depression, n (%)					0·28
No	266 (45·1)	69 (40·4)	117 (45·9)	80 (48·8)	
Yes	324 (54·9)	102 (59·6)	138 (54·1)	84 (51·2)	
Anxiety, n (%)					0·13
No	265 (44·9)	67 (39·2)	116 (45·5)	82 (50·0)	
Yes	325 (55·1)	104 (60·8)	139 (54·5)	82 (50·0)	
Health care professional, n (%)					0·14
No	276 (46·8)	76 (44·4)	131 (51·4)	69 (42·1)	
Yes	314 (53·2)	95 (55·6)	124 (48·6)	95 (57·9)	

Supplemental Table 3. Participant characteristics, stratified by region

Characteristics	Total	Regions						*p-value*
		North Central	North East	North West	South East	South South	South West	
	N=519	N=78	N=20	N=37	N=22	N=45	N=317	
BMI, M(±SD)	19·1 (3·3)	18·5 (2·7)	19·1 (1·9)	19·5 (3·4)	20·1 (4·2)	20·6 (3·3)	18·8 (3·4)	**0·006**
Gender, n (%)								**<0·001**
Male	203 (39·1%)	40 (51·3%)	12 (60·0)	29 (78·4)	8 (36·4)	23 (51·1)	91 (28·7)	
Female	312 (60·1)	36 (46·2)	8 (40·0)	8 (21·6)	13 (59·1)	21 (46·7)	226 (71·3)	
Missing	4 (0·8)	2 (2·6)	0 (0·0)	0 (0·0)	1 (4·5)	1 (2·2)	0 (0·0)	
Age Category, n (%)								0·16
<18-24 years	157 (30·3)	26 (33·3)	3 (15·0)	8 (21·6)	5 (22·7)	7 (15·6)	108 (34·1)	
25-29 years	243 (46·8)	37 (47·4)	13 (65·0)	20 (54·1)	9 (40·9)	25 (55·6)	139 (43·8)	
≥30 years	119 (22·9)	15 (19·2)	4 (20·0)	9 (24·3)	8 (36·4)	13 (28·9)	70 (22·1)	
Education Category, n (%)								0·54
<Bachelors	178 (34·3)	27 (34·6)	7 (35·0)	11 (29·7)	7 (31·8)	10 (22·2)	116 (36·6)	
≥Bachelors	341 (65·7)	51 (65·4)	13 (65·0)	26 (70·3)	15 (68·2)	35 (77·8)	201 (63·4)	
Employment Category, n (%)								**0·003**
Not Employed	242 (46·6)	43 (55·1)	13 (65·0)	23 (62·2)	12 (54·5)	26 (57·8)	125 (39·4)	
Employed	277 (53·4)	35 (44·9)	7 (35·0)	14 (37·8)	10 (45·5)	19 (42·2)	192 (60·6)	
Marital Status, n (%)								0·68
Married/Cohabitation/Common-law	114 (22·0)	17 (21·8)	4 (20·0)	7 (18·9)	5 (22·7)	12 (26·7)	69 (21·8)	
Separated/Divorced/Widowed	5 (1·0)	0 (0·0)	1 (5·0)	1 (2·7)	0 (0·0)	1 (2·2)	2 (0·6)	
Single	400 (77·1)	61 (78·2)	15 (75·0)	29 (78·4)	17 (77·3)	32 (71·1)	246 (77·6)	
Depression, n (%)								**0·025**
No	242 (46·6)	36 (46·2)	5 (25·0)	17 (45·9)	5 (22·7)	17 (37·8)	162 (51·1)	
Yes	277 (53·4)	42 (53·8)	15 (75·0)	20 (54·1)	17 (77·3)	28 (62·2)	155 (48·9)	
Anxiety, n (%)								**0·003**

No	231 (44·5)	35 (44·9)	4 (20·0)	10 (27·0)	6 (27·3)	16 (35·6)	160 (50·5)	
Yes	288 (55·5)	43 (55·1)	16 (80·0)	27 (73·0)	16 (72·7)	29 (64·4)	157 (49·5)	
Health care professional, n (%)								**<0·001**
No	257 (49·5)	59 (75·6)	18 (90·0)	29 (78·4)	17 (77·3)	33 (73·3)	101 (31·9)	
Yes	262 (50·5)	19 (24·4)	2 (10·0)	8 (21·6)	5 (22·7)	12 (26·7)	216 (68·1)	

Supplemental Table 4. Gender differences in the association between eHealth Literacy and Psychological outcomes

Psychological Outcomes	Male	Female
Depression	**0·44 (0·22-0·88)**	**0·27 (0·13-0·55)**
Anxiety	**0·32 (0·16-0·65)**	**0·33 (0·16-0·69)**
Fear of COVID-19	1·03 (0·45-2·34)	0·36 (0·11-1·24)

Bold: P<0·05

Adjusted for age, education, employment

Supplemental Table 5. Age differences in the association between eHealth Literacy and Psychological outcomes

Psychological Outcomes	18-24 years	25-29 years	≥30 years
Depression	0·59 (0·22-1·59)	**0·18 (0·07-0·42)**	0·50 (0·22-1·17)
Anxiety	0·48 (0·17-1·33)	**0·24 (0·11-0·53)**	**0·38 (0·16-0·91)**
Fear of COVID-19	0·28 (0·08-1·80)	0·84 (0·32-2·22)	0·70 (0·22-2·29)

Bold: P<0·05

Adjusted for gender, education, employment

Supplemental Table 6. Regional differences in the association between eHealth Literacy and Psychological outcomes

Psychological Outcomes	North Central	North East	North West	South East	South South	South West
Depression	**0·78 (0·58-0·99)**	0·80 (0·51-1·27)	**0·57 (0·32-0·99)**	0·90 (0·45-1·82)	**0·67 (0·49-0·91)**	**0·90 (0·82-0·98)**
Anxiety	0·79 (0·62-1·01)	0·83 (0·58-1·19)	**0·60 (0·42-0·86)**	0·73 (0·35-1·54)	**0·68 (0·51-0·93)**	**0·90 (0·83-0·99)**
Fear of COVID-19	**0·72 (0·56-0·92)**	--	1·03 (0·38-2·79)	0·83 (0·33-2·06)	1·02 (0·66-1·56)	0·92 (0·84-1·01)

Bold: P<0·05

Adjusted for age, gender, education, employment
